# Development of tetraculture spheroids as a versatile 3D model for personalized breast cancer research

**DOI:** 10.1038/s41598-025-12556-9

**Published:** 2025-07-28

**Authors:** Oliwia Piwocka, Karolina Sterzyńska, Agnieszka Malińska, Wiktoria M. Suchorska, Katarzyna Kulcenty

**Affiliations:** 1 Department of Electroradiology, Poznan University of Medical Sciences, Poznan, 61-866 Poland; 2 Doctoral School, Poznan University of Medical Sciences, Poznan, 60-812 Poland; 3 Radiobiology Laboratory, Department of Medical Physics, Greater Poland Cancer Centre, Poznan, 61-866 Poland; 4 Department of Histology and Embryology, Poznan University of Medical Sciences, Poznan, 61-781 Poland; 5 Department of Anatomy and Histology, Collegium Medicum, University of Zielona Gora, Zielona Gora, 65-046 Poland

**Keywords:** Tumor-microenvironment, Multicellular spheroids, Tumoroids, Breast cancer, 3D culture, Tetraculture, Personalized cancer research, Cancer, Breast cancer, Cancer models, Tumour heterogeneity, Cell biology, Cell signalling

## Abstract

The tumor microenvironment (TME) plays a pivotal role in cancer progression and drug resistance, influenced by the interaction of tumor cells with surrounding fibroblasts, immune, and endothelial cells. Developing robust multicellular tumor spheroids (MCTSs) that mimic the tumor microenvironment is crucial for studying cancer progression and therapeutic resistance. This study aimed to establish a reproducible method for generating MCTSs using a tetraculture system in four breast cancer cell lines: BT474, T47D, MDA-MB-231, and SK-BR-3. This approach incorporates primary cancer-associated fibroblasts (CAFs), macrophages (THP-1), and endothelial cells (Ea.hy926) alongside the cancer cells. MCTSs were generated using a simple method on ultra-low attachment plates, ensuring spheroid viability and uniformity across cell lines, confirmed by immunofluorescence and immunohistochemistry. MCTSs underwent extensive characterization, including invasion pattern analysis, macrophage polarization potential, cytotoxicity assay to assess chemotherapeutic resistance, and gene expression analysis to explore extracellular matrix (ECM) remodeling. The spheroids exhibited distinct morphologies, growth patterns, and cell distributions, reflecting unique microenvironment interactions and providing a reliable platform for studying TME. This versatile 3D model offers a promising platform for personalized therapy design, as it enables the incorporation of patient-derived cells regardless of tumor phenotype or inter-patient variability. Including key elements of the tumor microenvironment supports individualized drug testing and functional analysis, serving as a reproducible and ethically favorable alternative to animal models and patient-derived explants.

**Supplementary Information:**

The online version contains supplementary material available at 10.1038/s41598-025-12556-9.

## 1. Introduction

Personalized cancer therapy relies on models that reflect the tumour’s molecular characteristics and incorporate its dynamic microenvironment. Conventional 2D cultures and even some 3D systems often fail to recapitulate the complexity of tumor–stroma–immune interactions, limiting their translational potential. To overcome these challenges, there is an increasing focus on developing simplified, yet physiologically relevant, multicellular systems that allow functional testing of patient-derived material in a controlled setting.

The discovery of life-saving treatments would not be possible without preclinical studies that include research on cellular models and animals. This is a crucial step when scientists review their findings and decide whether the drug should be tested in people. Therefore, choosing an appropriate model to study cell-drug interactions is necessary. Three-dimensional (3D) models are becoming a gold standard in cancer research due to maintaining tumor microenvironment (TME) complexity, intracellular cross-talk, and similar conditions inside the tumor^1^. A two-dimensional (2D) model is the most common approach, however, it has some drawbacks, as 2D cell cultures do not mimic native structures, which might impact final research outcomes^2^. 3D platforms have been used to evaluate drug sensitivity, demonstrating greater resistance to chemotherapy and radiotherapy than cells cultured in 2D monolayers^3^. The distinct pharmacological responses seen in 2D and 3D systems might contribute to the high failure rate in drug discovery, as most preclinical cell-based screenings are conducted on 2D-cultured cells^4^.

The rapid development of 3D models, observed in recent years, proves the growing demand for accessible and simple protocols. 3D platforms comprise spheroids, organoids, and more sophisticated approaches like microfluidic systems, bioreactors, or tissue explants^5^. In the scientific literature, the term spheroid is broadly used to describe three-dimensional cell aggregates, but a variety of related terms, such as tumorospheres, mammospheres, and tumoroids, have also emerged to refer to specific subtypes that differ in cellular composition, origin, or culture conditions. For instance, mammospheres typically derive from mammary stem or progenitor cells under non-adherent conditions, whereas tumorospheres often refer to spheroids formed from cancer stem-like cells. Additionally, the term multicellular tumor spheroids (MCTSs) is variably applied to both monoculture and co-culture models, depending on whether a single cancer cell type or a mixture of tumor and stromal cells is used^6^. Poor standardization of nomenclature and various protocols to obtain spheroids might be confusing. Hence, Perisman and colleagues developed a MISpheroID platform providing methodological transparency and spheroid research guidelines. In nearly 88% of analyzed protocols for breast cancer (BC) spheroids, only 23.3% present data about spheroid morphology, whereas spheroid shape (i.e., circularity) is assessed in less than 1% of research. Spheroid morphology and size have a significant impact on the results. Considering this, MISpheroID establishes experimental conditions for spheroid studies, such as medium composition and spheroid morphology, which facilitate results interpretation and transparency^7^. Although the results presented by the MISpheroID Consortium were shown only for homospheroids, we will follow their guidelines in our paper.

Most studies establishing 3D models focus on a single cell line. However, homospheroids, composed solely of cancer cells, have certain limitations. They form compact cell aggregates that lack interaction with their extracellular environment, resulting in insufficient extracellular matrix (ECM) deposition and the absence of various stromal cell types^8^. The stromal interactions in 3D tumor models can affect drug penetration, therapeutic response, tumor progression, and multicellular resistance. When developing an in vitro cancer model, it is important to note that cancer cells behave differently when grown in co-culture conditions, as the cytokines and ROS levels vary^9^. The TME comprises diverse cell types, including cancer cells, fibroblasts, immune cells, and endothelial cells, all of which engage in dynamic and reciprocal interactions that influence tumor progression, therapeutic response, and metastatic potential^10^. Cancer-associated fibroblasts (CAFs) and macrophages play particularly prominent roles among these. CAFs secrete a wide range of cytokines, chemokines, and extracellular matrix components that modulate immune cell behavior, including the recruitment and polarization of macrophages^11^. In turn, tumor-associated macrophages (TAMs) shape the TME through their plasticity, exhibiting a spectrum of activation states often simplified into pro-inflammatory M1 and anti-inflammatory, tumor-promoting M2 phenotypes^12^. The interplay between CAFs and macrophages can reinforce immunosuppressive and pro-tumorigenic signaling loops, thereby supporting tumor invasion, angiogenesis, and resistance to therapy. Thus, monitoring M1/M2 polarization in such models serves not only as a marker of macrophage function but also as an indicator of the overall immunological state and therapeutic relevance of the TME^13^. Incorporating multiple stromal and immune components of the TME into in vitro cancer models ultimately increases their physiological relevance, robustness, and predictive value for therapeutic testing.

To our knowledge, only two papers describe the usage of tetracultures (MCTSs composed of 4 cell types). One model depicts pancreatic cancer (ancreatic, stellate, ECs, and monocytes)^14^ and another one lung cancer (alveolar type II cells, macrophages, mast and ECs)^15^. In this context, we have established a tetraculture spheroid model that incorporates four essential components of the breast tumor microenvironment: breast cancer cells (BC), cancer-associated fibroblasts (CAFs), endothelial cells (ECs), and macrophages. The protocol is compatible with representative cell lines for each of the four major molecular subtypes of breast cancer (including luminal A (T47D), HER2-enriched (BT474 and SK-BR-3), and triple-negative (MDA-MB-231) cell lines) and allows the incorporation of CAFs directly isolated from individual patients. The universality of the described method makes the model suitable for various tumor phenotypes and adaptable to patient-specific cell material. Ultimately, this system enables the establishment of 3D cultures that reflect the tumor microenvironment and can be used for further testing, such as evaluating drug sensitivity in a personalized setting. Compared to in vivo models involving xenografting of human cells into mice or patient-derived explants (PDEs), this approach offers a scalable, human-relevant alternative that preserves critical aspects of tumor biology while reducing reliance on animal experimentation.

## Results

### Dynamics of spheroid co-culture

Understanding the dynamics of multicellular tumor spheroids (MCTSs) is crucial for unraveling how different molecular subtypes of BC influence cellular organization, proliferation, and spheroid morphology. By analyzing the spatial distribution of cancer-associated fibroblasts (CAFs), macrophages, and endothelial cells within MCTSs, we can gain insights into subtype-specific interactions within the tumor microenvironment (TME), which are key drivers of tumor progression and therapeutic resistance.

**Fig. 1:**
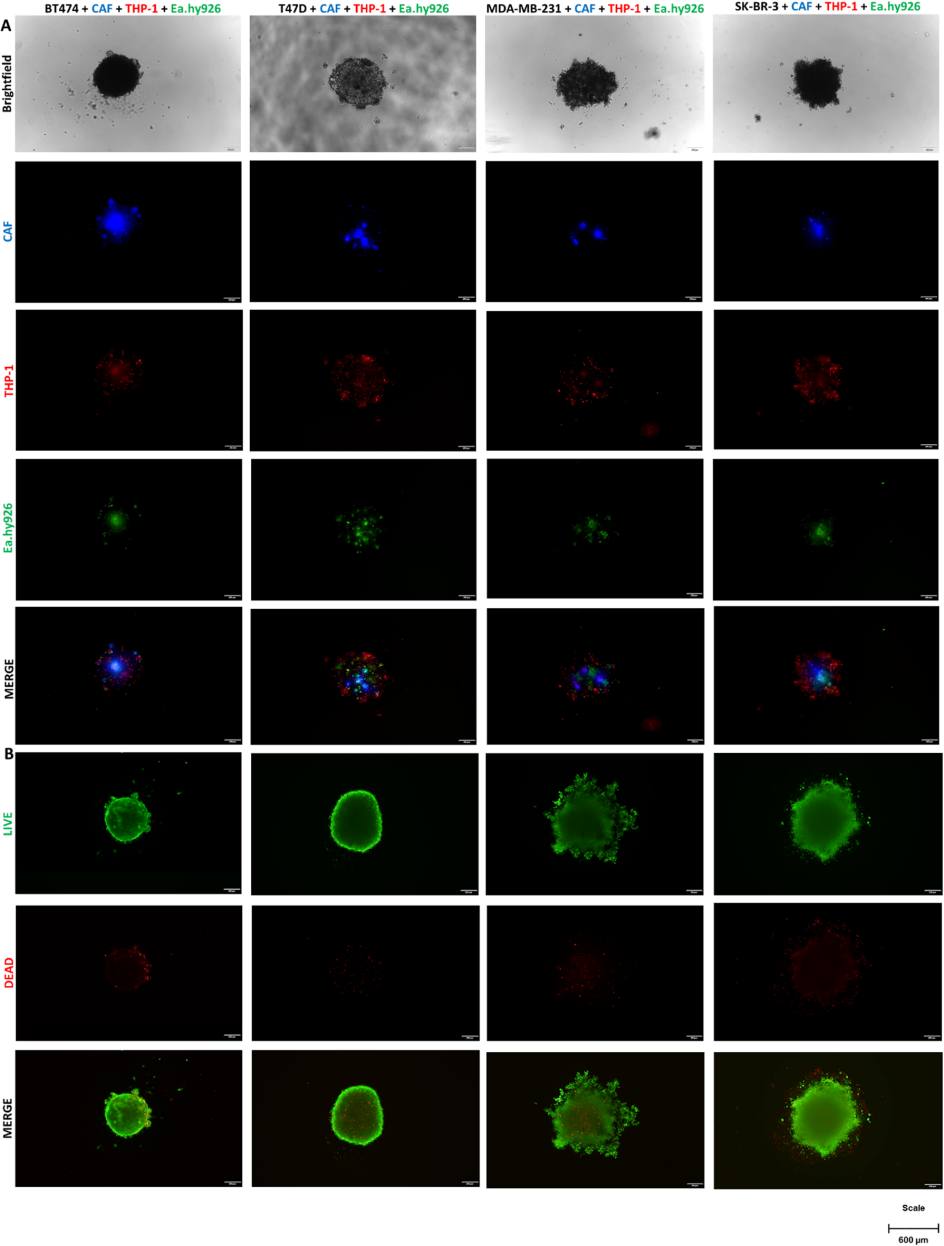
Immunofluorescently stained spheroids show various distributions of cells within the spheroid (green – Ea.Hy926, red – THP1, blue – CAFs; stacks of pictures with and without brightfield). The viability of generated MCTSs with different types of breast cancer cell lines (green – alive, red – dead) confirms high MCTSs viability. Magnification 4x, Olympus IX83 Inverted Fluorescence Microscope, scale 600 μm.

Immunofluorescent staining aimed to assess cellular distribution within MCTSs created with different types of cell lines (Fig. 1). CAFs (blue) tend to gather in the center of the BT474 and SK-BR-3 spheroids. In contrast, CAFs spread in clusters across spheroid in the T47D and MDA-MB-231 cell lines. Macrophages (red) are uniformly dispersed in BT474 and MDA-MB-231 MCTSs. In the case of SK-BR-3 and T47D, macrophages are also spread, however, they tend to direct to the outer layers of the sphere. ECs (green) are organized in smaller colonies in T47D, BT474, and MDA-MB-231 MCTSs but gather in one group near the center in SK-BR-3 MCTSs. Moreover, ECs prefer sites near the clusters of CAFs.

The MDA-MB-231 cell line has the largest spheroid area (386381 μm²), while the SK-BR-3 spheroids have the smallest (309006 μm²) (Table 1). Moreover, MDA-MB-231 spheroids have the largest diameter but the lowest circularity and roundness. Similar morphology and the manner of growth have SK-BR-3 spheroids. These spheroids form looser aggregates compared to BT474. Although they are not as tightly packed, they maintain their integrity and do not fall apart easily. This type of growth indicates a more spread-out cellular arrangement while maintaining overall structural stability. BT474 forms compact, well-defined, round aggregates, as indicated by the smallest diameter (568.76 μm) and the highest circularity (0.234). T47D spheroids show intermediate properties in terms of area, diameter, circularity, roundness, and they form cohesive spheroids.

Live/dead assay estimated the content of viable and dead cells within the spheres (Fig. 1). The test was performed after 7 days in culture, when MCTSs usually underwent other experiments. Spheres presenting more compact morphology, such as BT474 and T47D, had more dead cells in the center of the MCTSs, whereas spheroids composed of SK-BR-3 and MDA-MB-231 cell lines demonstrated higher content of dead cells in the outer layer of the MCTSs. After 7 days in culture, spheroids were primarily composed of viable cells with a small degree (up to 10%; Supplement Fig. 1) of dead cells.

**Table 1:** Morphological characteristics of given spheroids (mean values of 3 replicates and standard deviation).

Multicellular spheroid type	Area [µm^2^]	Diameter [µm]	Circularity	Roundness
BT474 + CAF +THP-1 + Ea.hy926	313,301 ± 5271,926	568.76 ± 16,400	0.234 ± 0,010	0.904 ± 0,018
T47D + CAF +THP-1 + Ea.hy926	332,132 ± 5049,110	692.25 ± 10,277	0.211 ± 0,009	0.902 ± 0,008
MDA-MB-231 + CAF +THP-1 + Ea.hy926	386,381 ± 6223,708	796.19 ± 18,460	0.167 ± 0,005	0.778 ± 0,014
SK-BR-3 + CAF +THP-1 + Ea.hy926	309,006 ± 6163,446	575.28 ± 11,084	0.206 ± 0,004	0.914 ± 0,004

**Fig. 2:**
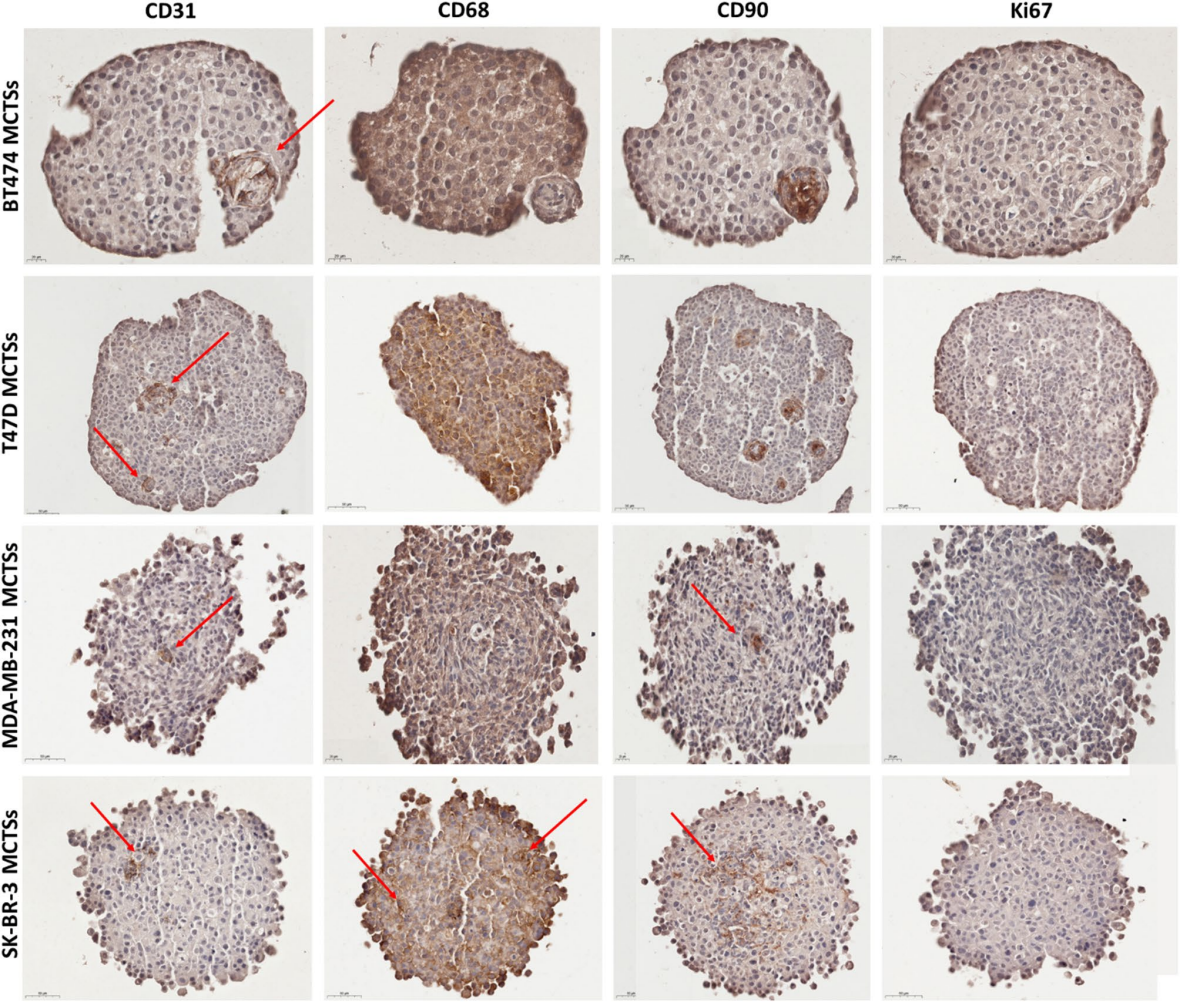
Immunohistochemical staining confirmed the presence of endothelial cells (CD31), macrophages (CD68), and CAFs (CD90). Ki67 was used to assess proliferation. ECs were detected in all models, most notably in T47D and SK-BR-3. CD68 staining indicated macrophage presence, with SK-BR-3 showing distinct positive cells (arrows). CD90 + CAFs formed clusters in BT474, MDA-MB-231, and T47D spheroids, and were also dispersed in the MDA-MB-231 core.

Immunohistochemistry (IHC) was conducted to confirm the presence of ECs, macrophages, and CAFs within the spheroids using cell-specific antibodies: CD31 for ECs, CD68 for macrophages, and CD90 for CAFs. Additionally, Ki67 was employed to assess cellular proliferation (Fig. 2). All cell lines exhibited some level of ECs, with the most noticeable presence observed in T47D and SK-BR-3 spheroids, where ECs were localized in smaller populations. Spheroids stained for CD68 emit dark signals throughout the sphere despite low antibody concentration, which hinders macrophage recognition. The specific and reliable indication of macrophages can be observed in the SK-BR-3 cell line, where a subset of cells is distinctly stained, serving as clear evidence of macrophage presence (indicated with arrows). CD90 staining revealed the presence of CAFs, which tended to cluster in distinct nests within BT474, MDA-MB-231, and T47D spheroids. In MDA-MB-231 spheroids, CAFs were also dispersed within the core. Ki67 staining, indicative of cellular proliferation, was generally sparse, suggesting low proliferative activity across all spheroid types.

### Multicellular spheroids present invasive potential

**Fig. 3:**
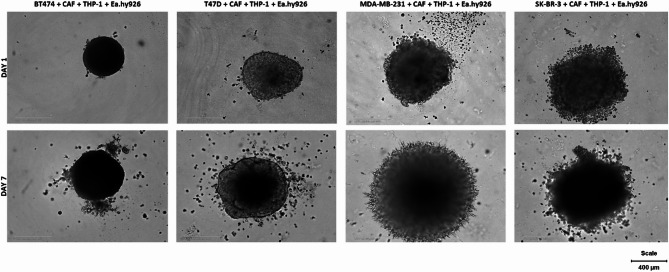
Spheroid invasion after 7 days. Cells were seeded according to the protocol and, after 5 days in culture, were embedded in 30% Matrigel. Invasion was observed for the subsequent 7 days. BT474 and MDA-MB-231 spheroids exhibited the most pronounced invasion, where BT474 formed individual protrusions, while MDA-MB-231 showed a diffuse, spiky invasion pattern. SK-BR-3 and T47D spheroids displayed less prominent invasion, characterized by scattered, dispersed cells around the spheroid periphery. Magnification 10x, scale 400 μm, Incucyte^®^ SX3 Live-Cell Analysis System.

Once cancer cells separate from the primary tumors, metastatic cells begin producing matrix metalloproteinases, which enable them to invade the extracellular matrix by breaking down the basal membrane. We investigated invasion properties in MCTSs in different cell lines. The most profound effect was visible in BT474 and MDA-MB-231 MCTSs. In the case of BT474, visible protrusions escape from the spheroid, creating distinct colonies and indicating significant cell movement away from the primary mass (Fig. 3). MDA-MB-231 MCTS has an irregular and fuzzy appearance, indicating a high cell migration and spreading level. On the other hand, T47D and SK-BR-3 MCTSs present different types of invasion with less elevated effects. After 7 days, T47D and SK-BR-3 spheroids have a more dispersed appearance and less compact structure, suggesting some level of invasion. The extent and type of invasion might be less pronounced or aggressive compared to other cell lines.

The analysis of invasive potential in MCTSs revealed distinct differences across BC cell lines. BT474 and MDA-MB-231 spheroids exhibited the highest invasive behavior, with BT474 forming prominent protrusions and MDA-MB-231 displaying irregular, fuzzy morphology indicative of significant cell migration. In contrast, T47D and SK-BR-3 spheroids showed less pronounced invasion, characterized by dispersed structures and reduced compactness, suggesting a lower but still notable invasive capacity.

### Macrophage polarization

**Fig. 4:**
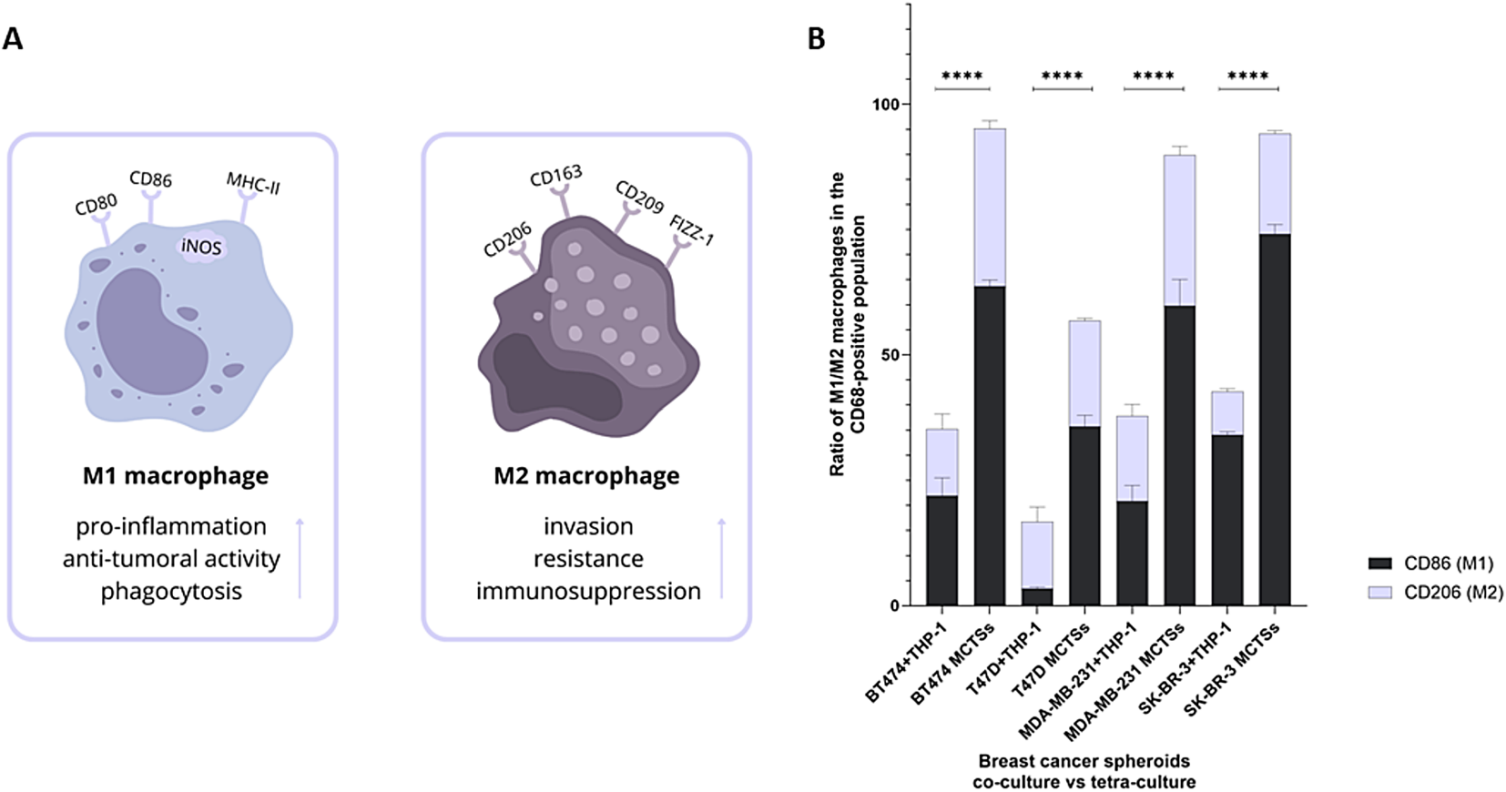
(**A**) M1 and M2 macrophage characteristic features and markers used for identification. M1 macrophages are associated with membrane expression of CD80, CD86, and MHC-II and cytoplasmic expression of iNOS. M2 macrophages are usually related to CD163, CD206, CD209, and FIZZ-1 markers. **(B)** A ratio of M1 and M2 macrophages in spheroid co-culture of cancer cells and macrophages (THP-1), and in MCTSs. M1/M2 content was measured by capturing one thousand CD68-positive cells (a marker for all types of macrophages) and capturing CD86/CD206-positive cells within the CD68 group. The number of M1 and M2 cells was divided by the number of CD68-positive cells to obtain a percent ratio of macrophage M1/M2 content. Macrophage phenotyping shows a significantly higher number of M1 and M2 macrophages in all types of MCTSs than in the co-culture of cancer cells with macrophages. *p* > 0.05 not significant (ns); *p* ≤ 0.05 (*); *p* ≤ 0.01 (**); *p* ≤ 0.001 (***); *p* ≤ 0.0001 (****).

Macrophage polarization is a common process occurring within TME, most profoundly with the participation of CAFs. It is marked by the transition from M1 (anti-tumorigenic) to M2 (pro-tumorigenic) macrophages or in reverse^10^. This experiment highlighted the importance of incorporating stromal cells into spheroids in advance to co-culture cancer cells with macrophages. Macrophage polarization was evaluated based on M1 (CD86) and M2 (CD206) marker expression. Spheroids composed of cancer cells co-cultured with macrophages (BT474 + THP-1, T47D + THP-1, MDA-MB-231 + THP-1, SK-BR-3 + THP-1), as well as full tetraculture MCTSs, were analyzed by flow cytometry. Results indicate that all types of MCTSs have significantly higher M1 and M2 macrophage content than a simple co-culture of cancer cells with macrophages, where the M2 fraction is meaningful due to its pro-tumorigenic features (Fig. 4).

The results demonstrate that macrophage polarization is significantly enhanced within the MCTSs compared to simple co-cultures of cancer cells and macrophages. These results suggest that incorporating stromal cells into MCTSs promotes both M1 and M2 macrophage populations, with a notable increase in the pro-tumorigenic M2 fraction. These findings highlight the critical role of the TME in driving macrophage polarization, underscoring the importance of using advanced co-culture models.

### Comparison of characteristics in 2D and 3D cell cultures

This experiment compared MCTSs to their 2D cell culture counterparts. Breast cancer cell lines were co-cultured with CAFs, THP-1 cells, and Ea.hy926 cells in a 4:1:1:1 ratio.

**Fig. 5:**
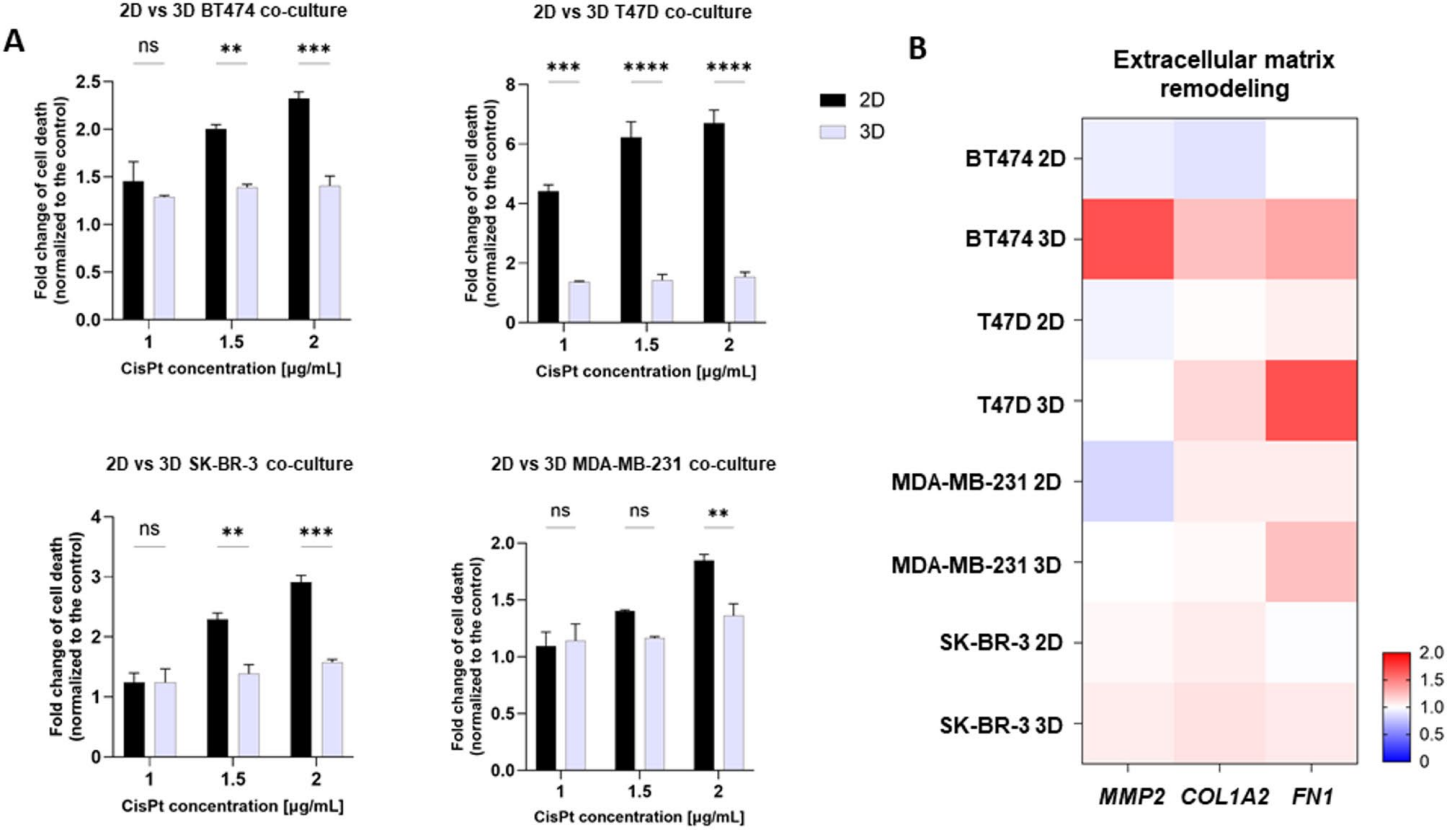
**(A)** Comparison of cell death in response to CisPt in 2D and 3D cell cultures; The graphs illustrate the fold change in cell death, normalized to the control, comparing 2D and 3D tetraculture models in response to CisPt treatment across four BC cell lines. For BT474, T47D and SK-BR-3, the extent of cell death is significantly lower in the 3D culture compared to the 2D culture across all tested CisPt concentrations. In contrast, for the MDA-MB-231 cell line, a significant reduction in cell death in 3D culture is observed only at the highest cisplatin concentration (2 µg/mL). These findings highlight the impact of culture dimensionality on cellular sensitivity to CisPt, with 3D culture conditions generally providing a more resistant phenotype. Representative images of 2D and 3D heterogenous cell cultures of SK-BR-3 cell line are provided in the Supplementary Fig. 2; **(B)** Genetic expression of ECM remodelinggenes (***MMP2***, ***COL1A2***, ***FN1***) in 2D and 3D setups. A fold change of gene expression was calculated with an expression equal to 1 being unchanged, < 1 indicating upregulation, > 1 indicating downregulation. The most noticeable changes were exhibited in BT474, T47D and MDA-MB-231 3D MCTSs with overexpression of *MMP2* and *COL1A2* for BT474 and *FN1* for MDA-MB-231 and T47D, presenting distinct mechanisms of ECM remodeling. *p* > 0.05 not significant (ns); *p* ≤ 0.05 (*); *p* ≤ 0.01 (**); *p* ≤ 0.001 (***); *p* ≤ 0.0001 (****).

Increased drug resistance in 3D cultures compared to 2D is a well-established occurrence in the literature. This section aimed to demonstrate that tetraculture MCTSs accurately reproduce this fundamental and clinically relevant feature of tumor biology. To compare the response of 2D and 3D cultures to cisplatin (CisPt) treatment, cell death was assessed using a live/dead assay. In the 2D cultures, the number of dead cells was quantified by counting red fluorescent objects, while in the 3D cultures, the mean intensity of red fluorescence was measured to account for the structural differences. Results were normalized to their respective untreated controls for both 2D and 3D cultures, allowing for the calculating of fold changes in cell death. Controls used in this study included 2D and 3D tetracultures not treated with CisPt. This approach enabled an objective comparison between the two culture systems, despite methodological differences inherent to the formats. The results demonstrate that 3D co-cultures are more resistant to CisPt treatment compared to their 2D counterparts across the tested BC cell lines. In the BT474, T47D, and SK-BR-3 models, significantly higher levels of cell death were observed in 2D cultures at increasing concentrations of CisPt, while 3D cultures showed reduced susceptibility, as indicated by lower fold changes in cell death (Fig. 5A). The MDA-MB-231 model exhibited a similar trend, although the difference became significant only at the highest concentration of cisplatin (2 µg/mL). Confirming resistance supports the physiological relevance of the model and validates its utility for future therapeutic screening and mechanistic studies.

Extracellular matrix (ECM) dynamics were analyzed using RT-qPCR to measure the expression of ECM remodeling genes (*MMP2*,* COL1A2*, and *FN1*). Fold changes in gene expression were calculated, with a value of 1 indicating no change, above 1 indicating upregulation, and below 1 indicating downregulation. The most profound changes were observed in the BT474, T47D and MDA-MB-231 models. In BT474 cultures, the differences between 2D and 3D setups were most striking for *MMP2*, which was downregulated in 2D but upregulated in 3D, suggesting active ECM degradation (Fig. 5B). Similarly, *COL1A2* and *FN1* was upregulated in 3D but downregulated in 2D. For MDA-MB-231 cells, *FN1* exhibited a slight increase in 3D cultures. T47D cells showed increased expression in *COL1A2* and *FN1* compared to 2D cells. In contrast, SK-BR-3 cells exhibited only slight variations in all genes between 2D and 3D setups. However, despite the observed trends, these differences were not statistically significant. These findings align with the invasion capabilities of each cell line, as BT474 and MDA-MB-231 showed the most significant invasion (Fig. 3), whereas SK-BR-3 and T47D MCTSs demonstrated modest invasion. The differences in ECM gene expression may reflect distinct mechanisms of ECM remodeling and invasion strategies used by these cell lines.

## Discussion

The tumor microenvironment (TME) is crucial in cancer progression, metastasis, and therapeutic resistance. It consists not only of cancer cells but also a variety of stromal components, including fibroblasts, endothelial cells, and immune cells, all of which engage in dynamic interactions that shape tumor behavior. Understanding these complex behaviors is essential for developing more effective therapeutic strategies. While animal models have historically served as a platform for studying tumor biology and drug response, their translational relevance is often limited by interspecies differences in immune responses, stromal composition, and drug metabolism^16^. To address these limitations, advanced in vitro 3D cancer models have emerged as powerful tools that more accurately recapitulate human tumor architecture and cell-cell interactions. Spheroid models allow for the incorporation of multiple patient-specific tumor and stromal cells, and offer the advantage of precise control over experimental variables, which enables dissection of therapeutic responses in a human-relevant context^17^. In this study, we present a robust method for generating viable and responsive breast cancer spheroids that incorporate key stromal components—cancer-associated fibroblasts (CAFs), macrophages, and endothelial cells closely mimicking the complexity of the TME. This approach is adaptable across multiple molecular subtypes of breast cancer, including luminal A (T47D), HER2-enriched (BT474 and SK-BR-3), and triple-negative (MDA-MB-231) cell lines. By integrating patient-derived stromal cells, our model holds significant potential for personalized medicine, supporting individualized drug screening and preclinical therapeutic testing dedicated to specific tumor profiles. Tetraculture spheroids offer distinct advantages over explant-based systems, which are often constrained by limited tissue availability, viability, and lack of scalability^5^. In contrast to animal models, our 3D multicellular spheroids (MCTSs) provide a faster, more adaptable, and less invasive alternative that aligns with ethical considerations and allows for high-throughput applications. By accurately recreating aspects of human tumor heterogeneity in a controlled, reproducible environment, this system represents a valuable tool for translational oncology and TME research.

Spheroids are cell clusters over 200 µM in diameter, featuring nutrients and oxygen gradient within the sphere^3^. Spheroid size is crucial since large spheroids tend to create a necrotic core and spheroids above 700 µM may be destroyed^8^. With this information, we carefully chose the number of cells to obtain viable spheroids on days 5–7 that may be subjected to experiments. Moreover, a ratio of seeded cells is vital as cells have distinct morphology and exist in the TME at various concentrations. To ensure physiological relevance, the cell ratios used in our spheroids were informed by spatial transcriptomic data from tumors, especially breast cancer, which allowed us to approximate the proportion of cancer cells to various TME components based on their in situ distribution^18–22^. Another critical factor is incorporating immune cells into the spheroids to create an immunogenic niche. Among immunocytes, the most profound population in the TME are macrophages, which play a crucial role in intracellular signaling due to their various phenotypes, such as M1 and M2 niches (Fig. 4A)^23^. Lastly, including ECs in a 3D model is crucial to reflect physiological conditions, such as vessel formation. Moreover, combining macrophages with ECs seems pivotal, as the immunomodulatory role of ECs has proven to be a crucial factor in shaping the immune response^24^.

There are many approaches for 3D cell culture, among them, the simplest way is to prevent cells from attaching to the culture vessel. Those methods include a hanging-drop method and culture on ultra-low attachment plates or wells covered in the matrix. More sophisticated approaches are microfluidic cultures, lab-on-chip or bioprinting^25^.We aimed to formulate an easy method for MCTSs establishment that would be uniform and suitable for different cell lines and would not involve additional/sophisticated devices. To confirm the stability and viability of spheroids, we conducted immunofluorescence, immunohistochemistry, and live/dead assay. Moreover, we demonstrated that key hallmarks of tumor behavior, such as invasive capacity, ECM remodeling, and increased resistance to chemotherapy, are preserved in our MCTSs. These fundamental features were confirmed through functional assays, including invasion assays, evaluation of macrophage polarization as an example of intercellular cross-talk, and drug response analysis comparing 3D spheroids to their 2D counterparts.

Immunofluorescent cell tracking is a powerful tool to explore spheroid properties. For accurate visualization various microscopy techniques are employed including inverted microscopes, confocal microscopy and more sophisticated methods i.e. light-sheet microscopy^26^. The immunofluorescent staining showed distinct patterns of cellular distribution among the spheroids. CAFs predominantly centralized within BT474 and SK-BR-3 spheroids, while they were more diffusely distributed in T47D and MDA-MB-231 spheroids (Fig. 1). This suggests a possible difference in the interaction mechanisms of CAFs with these cancer cell lines, or it is an individual feature of each cell line. Macrophages exhibited a more uniform distribution in BT474 and MDA-MB-231 MCTSs, but localized towards the outer layers in SK-BR-3 and T47D spheroids. ECs prefer to cluster near CAFs^18^ which could indicate a supportive role of CAFs in promoting vascular structures within the spheroids^27^. Regarding the spheroid inner core, recent literature reports that in the co-cultures of epithelial and stromal cells, epithelial cells tend to localize at the periphery due to their polarity, while stromal cells provide structural support and remain within the spheroid core^28^. A similar distribution pattern has also been observed in vivo, as well as, in our MCTSs model. Moreover, prevailing conditions within the spheroids include hypoxia, oxygen and nutrient gradient, which shifts highly proliferating cells outside and slowly-proliferating are maintained inside^29^.

Morphologically, MDA-MB-231 spheroids were the largest but least circular and round, indicating a more irregular growth pattern (Table 1). Although SK-BR-3 spheroids have the smallest area, they share a similar loose aggregate structure with MDA-MB-231. It is worth noting that spheroid integrity varies across different breast cancer cell lines, reflecting their intrinsic biological characteristics. In the MCTSs model, SK-BR-3 and MDA-MB-231 cells formed less compact spheroids compared to BT474 or T47D. However, this does not imply structural fragility. Despite their morphology, SK-BR-3 and MDA-MB-231 spheroids remained cohesive and could be handled by pipetting or transferring without disintegration. Although their outer rim appeared more susceptible to mechanical disruption, the core structure remained intact, allowing for successful completion of multiple downstream assays. This variability mirrors the diversity observed in tumor architecture in vivo and underscores the physiological relevance.

The live/dead assay highlighted a correlation between spheroid compactness and cell viability (Fig. 1). More compact spheroids (BT474 and T47D) had higher central cell death, possibly due to limited nutrient and oxygen diffusion^30^. Conversely, SK-BR-3 and MDA-MB-231 spheroids, with their looser structures, showed higher peripheral cell death. Despite these variations, all spheroids primarily consisted of viable cells after 7 days, indicating successful maintenance of overall spheroid viability. Ki67 staining revealed low proliferative activity across all spheroid types, which might reflect the established equilibrium in cellular proliferation and death within mature MCTSs. The presence of ECs, macrophages, and CAFs within the spheroids was confirmed via immunohistochemistry, validating the co-culture system and the role of these cells in mimicking the tumor microenvironment (Fig. 2). The SK-BR-3 cell line appears to be the most challenging to culture among BC cell lines. Frohlich et al. assessed 42 different setups for spheroid generation and found that SK-BR-3 cells did not form spheres under any conditions^31^. Falkenberg and colleagues cultured different BC cell lines by the hanging drop method, but SK-BR-3 did not form spheroids^32^. Another research group cultured SK-BR-3 mammospheres on 6-well plates coated in poly-2-hydroxyethyl-methacrylate; however, those cells easily formed disruptive aggregates rather than spheres^33^. Other groups successfully cultured SK-BR-3 on microchips^26^ or in fluid flow/pressure systems^34^. Moreover, the co-culture of cancer cells with other TME cells may facilitate spheroid formation due to the production of ECM components^35^ which might be a reason why SK-BR-3 cell lines in our model grow easily and form cohesive spheroids.

A crucial part of investigations within TME is intercellular cross-talk. Cancer cells, stromal and immune cells constantly exchange signals via secreted cytokines or exosomes, causing changes in phenotypes, vasculogenesis, and cancer progression^36^. Heterogenous spheroids are suitable models to study intercellular cross-talk, hence, we intended to show the relevance of incorporating different types of cells into spheroids based on macrophage polarization. Macrophage polarization is the process by which macrophages acquire a specific functional phenotype (M1 or M2) in response to stimuli from the microenvironment^37^. M1 macrophages secrete cytokines that suppress the proliferation of nearby cells and cause damage to surrounding tissue, whereas M2 macrophages produce cytokines that stimulate cell proliferation and support tissue repair^13^. Macrophage phenotyping was performed in spheroid co-culture of cancer cells (BT474, T47D, MDA-MB-231, SK-BR-3) and THP-1 cells, and tetracultures of MCTSs. Flow cytometry results indicate a significant increase in M1 and M2 macrophages in all types of MCTSs compared to co-culture of cancer cells and macrophages only (Fig. 4). MCTSs contain CAFs, which secrete proteins such as interleukins, CXCL12, and SDF-1 that may induce macrophage polarization^10^. Therefore, elevated M1/M2 macrophage fraction could be observed compared to spheroids without CAFs, underscoring the importance of spheroids diversification and enrichment.

The investigation into invasive behaviors demonstrated distinct capabilities among the spheroid models. BT474 and MDA-MB-231 spheroids exhibited significant invasive potential, with BT474 showing clear protrusions and colony formation and MDA-MB-231 displaying high cell migration and spreading (Fig. 3). This aligns with the aggressive nature of these cell lines in clinical settings^38^. In contrast, T47D and SK-BR-3 spheroids displayed less pronounced invasion, suggesting a more contained or less aggressive invasive phenotype. Additionally, ECM remodeling gene expression was evaluated compared to 2D cell cultures. ECM remodeling is crucial in tissue development, wound healing, and cancer progression. In the context of cancer, ECM remodeling supports tumor growth, metastasis, and drug resistance by altering the tumor microenvironment^39^. The key genes involved in ECM remodeling are typically responsible for degrading or reorganizing components of the ECM, i.e., matrix metalloproteinases (*MMP2*, −9, −14), disintegrins^39^ lysyl oxidases, and transglutaminases^40^. We analyzed the expression of *MMP2*, *COL1A2*, and *FN1* to assess the ability of 2D and 3D cell cultures to remodel ECM. *MMP2* is involved in ECM degradation, primarily breaking down type IV collagen, a key basement membrane component. Its upregulation can enhance invasive behavior, as cells must degrade ECM barriers for migration and metastasis^39^. *MMP2* is highly upregulated in BT474 3D, indicating a role in ECM remodeling in this condition (Fig. 5B). In the case of the MDA-MB-231 cell line, mild downregulation in 2D was observed. Contrary to our results, other research groups found increased activity of MMPs in MDA-MB-231 co-cultures with normal fibroblasts and CAFs^41^. *MMP2* expression is minimal in 2D and 3D cell cultures of T47D and SK-BR-3, suggesting that those cells do not heavily rely on *MMP2* for ECM remodeling, aligning with their relatively non-invasive behavior. *COL1A2* encodes part of type I collagen, one the most abundant forms of collagen in the ECM^42^. Increased collagen production can lead to a stiffer ECM, promoting tumor progression and resistance^43^. *COL1A2* expression is modest in BT474 and T47D, suggesting a role in collagen production. SK-BR-3 cultures show minimal expression, indicating a lack of involvement in collagen-mediated ECM stiffening. *FN1* is a key ECM glycoprotein involved in cell adhesion, migration, and wound healing. It plays a role in cancer cell motility and metastatic processes, especially in 3D environments where cells interact more dynamically with the matrix^44^. In our experiment, 3D cell cultures of BT474, T47D and MDA-MB-231 3D shows the highest upregulation of *FN1*, aligning with its role in promoting cell motility and invasion in metastatic cells. Park and Helfman demonstrated that elevated *FN1* levels in 3D suspension culture enhance cancer cell adhesion and spreading through integrin β−5 and Src, indicating that the increased *FN1* expression supports the initial attachment of cancer cells to secondary sites following circulation during metastasis^45^. Feng et al. found that the transcription factor CREB3L1 mediates the expression of genes related to ECM remodeling, especially *COL1A1*,* COL1A2*,* FN1*. The study demonstrated that CREB3L1 is essential for the invasive and metastatic capabilities of MDA-MB-231 cells, which are a model for mesenchymal triple-negative BC. In contrast, BT474 cells, which represent a different subtype of BC, did not exhibit the same dependency on CREB3L1 for invasion and metastasis^46^. A remodeled ECM can limit the ability of drugs to penetrate the tumor, contributing to chemoresistance due to increased collagen deposition or changes in ECM stiffness^47^. Another essential process, matrix stiffening, arises from ECM remodeling. This process is driven by enzymes such as MMPs, the lysyl oxidase (LOX) family, and the Procollagen-Lysine,2-Oxoglutarate 5-Dioxygenase (PLOD) family and is closely related to therapy resistance^48^.

Cells in monolayer cultures are uniformly exposed to chemotherapeutic agents and are primarily composed of proliferative cells. Consequently, 3D cell culture models, which include cells at different cell cycle stages, tend to be more resistant to chemotherapy than traditional 2D cultures. This resistance reflects in vivo conditions more accurately, including cell cycle diversity, cell morphology, nutrient requirements, and cellular behavior^49^. Higher resistance may result from changes in receptor proteins, drug transporters, and enzyme activity involved in drug metabolism. Świerczewska and colleagues assessed the response of 6 chemotherapeutic drugs in 2D and 3D cell cultures of ovarian drug-sensitive and resistant cell lines. They found that the effectiveness of drugs in spheroids is influenced by the spheroid size and structure (dense or loose), the type of cells (necrotic, quiescent, or proliferating), drug concentrations, the ability of the drug to diffuse through the ECM. Moreover, results indicated that drug-sensitive and drug-resistant cells cultured in 3D demonstrate greater resistance than those cultured in 2D^50^. The results underscore the necessity of incorporating 3D culture methods alongside 2D approaches in preclinical studies for new targeted therapies and traditional anti-cancer drugs^51^. Hence, we performed a study to assess cell death in the response to CisPt treatment in 2D versus 3D heterogeneous cell cultures. Cytotoxicity was evaluated by fluorescent labeling of unviable cells. 3D cultures exhibited higher resistance in our settings than 2D cultures. Additionally, 3D cultures showed lower response levels when CisPt concentration was increased.

In conclusion, this study underscores the importance of 3D MCTSs as advanced in vitro models that closely mimic the complexity of the TME. By incorporating key stromal components, such as CAFs, ECs, and macrophages, we observed distinct spatial organization, enhanced stability, and high viability of spheroids. The ability of CAFs to influence macrophage polarization was evident, as MCTSs exhibited significantly elevated M1 and M2 macrophage fractions compared to simple co-cultures, highlighting the critical role of intercellular signaling within the TME. Furthermore, invasive properties varied across cell lines, with BT474 and MDA-MB-231 spheroids displaying significant invasion potential, while T47D and SK-BR-3 spheroids remained less aggressive, reflecting their respective clinical behaviors. Analysis of ECM remodeling genes (*MMP2*, *COL1A2*, and *FN1*) demonstrated upregulation in 3D cultures, particularly in BT474 and MDA-MB-231 spheroids, emphasizing their enhanced matrix interaction and potential for invasion. Importantly, chemoresistance assay revealed higher resistance in 3D MCTSs and higher susceptibility to CisPt treatment in 2D model, as evidenced by increased cell death in 2D cultures, aligning with the physiological drug resistance observed in vivo. These findings collectively highlight the value of 3D MCTS models for studying tumor-stroma interactions, invasion dynamics, ECM remodeling, and therapeutic responses, providing a more reliable platform for preclinical cancer research and drug testing.

## Conclusions

3D culture systems allow researchers to recreate human organs and diseases in one dish and thus hold great promise for many applications, such as regenerative medicine, drug discovery, precision medicine, and cancer research. Our findings underscore the importance of considering the heterogeneity of TME in cancer research. The differential behavior of spheroids depending on the cell line used highlights the need for personalized approaches in cancer treatment. Understanding the specific interactions between cancer cells and stromal components can inform the development of targeted therapies that disrupt these interactions to inhibit tumor growth and invasion. Future studies could focus on elucidating the molecular mechanisms underlying the observed differences in cellular distribution, viability, and invasion. Additionally, exploring the impact of various therapeutic agents on these spheroid models could provide insights into their efficacy and potential resistance mechanisms in a more physiologically relevant context.

## Materials and methods

### Cell culture

T47D, BT474, SK-BR-3, MDA-MB-231, Ea.Hy926, THP-1 cell lines were purchased from ATCC. T47D and BT474 were cultured in RPMI-1640 (Merck Millipore Corporation, Germany) medium with 10% fetal bovine serum (FBS) and 1% penicillin/streptomycin (P/S) (Merck Millipore Corporation, Germany). SK-BR-3, MDA-MB-231 and Ea.Hy926 were maintained in DMEM with 10% FBS and 1% P/S. THP-1 cells were cultured in RPMI-1640 with 10% FBS and 0.05 mM 2-mercaptoethanol.

### Isolation of primary cells

CAFs were derived from BC biopsy specimens, as described elsewhere^46^. Briefly, breast cancer biopsy tissue was minced into small fragments of approximately 1 mm³ and incubated overnight in 1 mL of digestion medium containing of DMEM supplemented with 1% penicillin/streptomycin, 0.14 mg/mL hyaluronidase (Thermo Fisher Scientific, France), and 1.6 mg/mL collagenase IV. Following digestion, the cell suspension was transferred into a tube containing 2 mL of PBS and centrifuged at 700 g for 5 min at room temperature. The supernatant was discarded, and the resulting pellet was resuspended in fresh culture medium and plated across three wells of a 12-well plate. Culture medium for primary CAFs consisted of DMEM/F12 (Biowest, France), supplemented with 20% fetal bovine serum (FBS) (Biowest, France), 10 ng/mL epidermal growth factor (EGF) (Sigma-Aldrich, MO, USA), 2 mM L-glutamine (Biowest, France), 0.5 µg/mL hydrocortisone (Biowest, France), 100 U/mL insulin (Bioton S.A., Poland), 1% P/S (Merck Millipore Corporation, Germany), and 0.5% amphotericin (Biowest, France). After 4–5 passages, a DMEM medium with 10% FBS and 10 ng/mL EGF was used to maintain the cell culture. CAFs underwent characterization to confirm their origin as described elsewhere^36,52,53^ and primary cell lines from these studies were included in this paper. Ethical approval for this study (approval no. 283/21) was obtained from the Bioethics Committee of Poznan University of Medical Sciences. All research involving human participants was conducted in accordance with the Declaration of Helsinki and relevant institutional guidelines and regulations. Informed consent was obtained from all participants involved in the study or their legal guardians.

### Monocytes differentiation

Monocyte cell line THP-1 were differentiated into macrophages by treatment with 10 ng/mL of phorbol 12-myristate 13-acetate (PMA) in growth medium. The next day, the medium containing PMA was changed to fresh growth medium, and on the subsequent day, macrophages were ready for further experiments or seeding within spheroids.

### Spheroid growth medium

Growth medium for spheroids composed of DMEM/F12 medium (Biowest, France), 1% P/S (Merck Millipore Corporation, Germany), 500 ng/mL hydrocortisone (Biowest, France), 10 ng/mL EGF (Sigma-Aldrich, MO, USA), FGF-2 (Sigma-Aldrich, MO, USA), and 1X B-27 (Thermo Fisher Scientific, MA, USA). The medium was changed every 2–3 days.

Although some cell lines in 2D were cultured in RPMI-1640, we decided to proceed with DMEM/F12 medium when culturing spheroids. This rationale stems from optimization process, which shows worse spheroid formation when cultured in RPMI-1640 (Fig. 2S).

### Spheroid generation

Spheroids were generated using ultra-low attachment (ULA) 96-well plates (Corning, NY, USA). On the same day, all four cell types - breast cancer cells (BT474, T47D, MDA-MB-231, SK-BR-3), primary CAFs, Ea.hy926 endothelial cells (ECs), and THP-1 macrophages - were seeded together to initiate co-culture conditions. Cancer cells were seeded at a density of 4000 cells per well, while each stromal cell type (CAFs, ECs, and macrophages) was added at 1000 cells per well, resulting in a total of 7000 cells per spheroid. Spheroids were maintained under standard culture conditions, and after 5–7 days of incubation, well-formed multicellular tumor spheroids (MCTSs) were obtained and used for downstream experiments.

### Viability

MCTSs viability was assessed with LIVE/DEAD™ Viability/Cytotoxicity Kit (Thermo Fisher Scientific, MA, USA). A staining solution was prepared by adding 5 µL of calcein AM and 20 µL ethidium homodimer-1 to 10 mL PBS. The growth medium was removed from the wells, and 200 µL of the staining solution was added directly to the cells. Spheroids were incubated for 30 min at 37 °C in the dark, and then immunofluorescence was observed with Olympus IX83 Inverted Fluorescence Microscope (Olympus, Japan).

### Apoptosis

Spheroids from 36 wells were singularized with trypsin-EDTA solution (Thermo Fisher Scientific, MA, USA) and incubated for 15 min on a shaking incubator at a speed of 300 and 37 °C. Cell solution was then filtered through a 70 μm cell strainer (Corning, NY, USA). Apoptosis was assessed with ApoFlowEx FITC Kit (ExBio, Czech Republic). Cells were pelleted and resuspended in 10x Annexin Binding Buffer with 5 µL of Annexin V and 5 µL of propidium iodide and incubated for 15 min in the dark at room temperature. Then, samples were centrifuged and suspended in the buffer for further assessment. Analysis was performed on the Cytoflex Beckmann Coulter cytometer (Beckman Coulter Life Sciences, ID, USA). The results were analyzed using FlowJo v10 (FlowJo LLC, USA).

### Immunofluorescence

Cells were stained with PKH67 Green Fluorescent Cell Linker Kit, PKH26 Red Fluorescent Cell Linker Kit, and BioTracker 400 Blue Cytoplasmic Membrane Dye (Merck Millipore Corporation, Germany). Ea.Hy926 was stained with PKH67 and THP-1 with PKH26. Cells were trypsinized, neutralized with growth medium, and centrifuged at 400 g for 5 min. Then, the pellet was washed with medium without FBS and centrifuged again. The staining solution was prepared by adding 4 µL of PKH67 or PKH26 stain to 1 mL of the Diluent C. Cell pellet was resuspended in 1 mL of the Diluent C, and 1 mL of staining solution was added. Cells were incubated for 4–5 min and staining was inhibited with 2 mL of 1% BSA/PBS. Afterward, cells were centrifuged at 400 g for 10 min. Then, cells were suspended in a growth medium and centrifuged twice at 400 g for 5 min. CAFs were stained with BlueTracker. The labeling solution was prepared by mixing 5 µL of the Cell Labeling Solution and 5 µL of the Loading Buffer in 1 mL of growth medium. Cells were collected into the tube, resuspended in labeling solution, and incubated for 20 min at 37 °C. Then, cells were pelleted by centrifugation at 1500 rpm for 5 min, resuspended in a growth medium, and centrifuged twice. After staining, all cells were seeded as described above. After 7 days, immunofluorescence was observed with Olympus IX83 Inverted Fluorescence Microscope.

### Invasion

After 5 days of MCTSs in culture, an invasion assay was performed. The growth medium was gently removed. Subsequently, 100 µL of a 30% Matrigel solution, prepared by diluting growth factor-reduced Matrigel (Corning, NY, USA) in additive-free DMEM/F12 medium, was added to each well. The Matrigel was kept on ice and handled gently to prevent premature polymerization. Following addition, plates were incubated at 37 °C for 30 min to allow the matrix to solidify. Spheroid invasion into the surrounding matrix was monitored for 7 days using the Incucyte^®^ SX3 Live-Cell Analysis System (Sartorius, Poland), with images acquired using a 10x objective.

### FFPE sections preparation

Spheroids were collected from 12 wells and placed in a 1.5 mL centrifuge tube, centrifuged at 200xg for 5 min and then the medium was replaced with PBS and centrifuged again. The supernatant was discarded and replaced with 1 mL of 4% paraformaldehyde for 30 min incubation. The spheroids were then washed three times with PBS and dehydrated in a series of ascending alcohol solutions (70%, 85%, 96%, and 100%) for 10 min each. They were then incubated in xylene twice for 10 min and centrifuged at 200xg for 5 min. The xylene was removed, and the spheroids were embedded in melted paraffin. After the paraffin had solidified, the tip of the tube containing the spheroids was cut off, placed on the embedding mold, and filled with melted paraffin. The paraffin blocks were sectioned at 4 μm using a semi-automatic rotary microtome (Leica RM 2145, Leica Microsystems, Nussloch, Germany).

### IHC staining

The spheroid sections were deparaffinized in xylene for 5 min, twice, and hydrated in a series of descending alcohol concentrations (100%, 96%, 85%, and 70% for 2 min each). The sections were then blocked in normal goat serum for 30 min and incubated with a specific primary antibody overnight at 4 °C, followed by incubation with the EnVision Detection System (Dako REALTM EnVisionTM Detection System peroxidase/DAB+, Rabbit/Mouse, Dako, Glostrup, Denmark). The following primary antibodies were used: mouse monoclonal anti-CD31 (PECAM-1) (1:1000; #3528; Cell Signaling Technology, Danvers, MA, USA), mouse monoclonal anti-CD68 (1:400; #11–749; Exbio, Vestec, Czech Republic), mouse monoclonal anti-CD90 (1:200; #10–652; Exbio, Vestec, Czech Republic), mouse monoclonal anti-Ki67(1:200; #11–155; Exbio, Vestec, Czech Republic). Finally, the slides were counterstained with hematoxylin (#S330930-2, DAKO, CA, USA), dehydrated and mounted with the Shandon Consul-Mount Histology Formulation histofluid (Thermo Fisher Scientific, Massachusetts, USA) and coverslips. All spheroid sections were digitized using a Grundium Ocus^®^20 Microscope Scanner (Tampere, Finland). Analysis of documented IHC staining was performed using CaseViewer 2.3 (64-bit version) for Windows (3D Histech Ltd., Budapest, Hungary).

### Macrophage phenotyping

Macrophage polarization was assessed in a simple co-culture of spheroids consisting of cancer cells and macrophages only and in MCTSs. To create a co-culture of cancer cells and macrophages, 4000 cells of BT474, T47D, MDA-MB-231, and SK-BR-3 were seeded with 1000 THP-1 cells. MCTSs were seeded as described above. Spheroids from 36 wells were harvested, singularized with trypsin and filtrated through 70 μm cell strainer. Trypsin was neutralized with growth medium and cell suspension was centrifuged at 1200 rpm for 5 min. Pellet was suspended in PBS and washed twice. Cells were incubated for 30 min at 4 °C with 5 µL of the following antibodies: CD68 (cat. number: 1P-749-T100), CD86 (cat. number: 1 A-531-T100) and CD206 (cat. number: 1P-782-T100) (Exbio, Vestec, Czech Republic). Afterward, cells were washed once with PBS. Stained cells were analyzed using a Cytoflex Beckmann Coulter cytometer (Beckman Coulter Life Sciences, ID, USA). The results were analyzed using FlowJo v10 (FlowJo LLC, USA).

### RT-qPCR

Cells were harvested in 500 µL TRI Reagent (Sigma Aldrich, Missouri, USA), and RNA was extracted by phenol/chloroform method. Extracted RNA was measured and evaluated for any impurities on the NanoDrop spectrophotometer (DeNovix, Delaware, USA) and by electrophoresis. The reverse transcription was performed using the RevertAid First Strand cDNA Synthesis Kit (Thermo Fisher Scientific, Massachusetts, USA). The cDNA was amplified in a total volume of 20 µL and diluted 5 times. Next, the expression of genes was analyzed using RT-qPCR. Genetic expression of the following genes was evaluated: *B2M*,

*ACTB*, Fibronectin (*FN1*) (Sigma Aldrich, Missouri, USA), *MMP2*, *COL1A2* (Roche Holding, Basel, Switzerland). Gene expression levels were calculated relative to the expression of a validated reference genes B2M and ACTB. The results represent mean values obtained from three independent biological replicates and compared between 2D vs. 3D culture. Primers’ sequences were listed in the Supplementary Table 1. The PCR reaction was conducted in the CFX96 Touch Real-Time Detection System (Bio-Rad Hercules, CA, USA) in 10 µL volume.

### Cell death in response to the treatment

Cells cultured in 2D and 3D were treated with 1, 1.5, and 2 µg/mL of cisplatin (Merck Millipore Corporation, Germany). Spheroids were seeded as described above, and co-culture of cells in 2D was prepared respectively to the 3D culture and seeded in ratio of 4:1:1:1 on 48-well plates. Cisplatin treatment was applied for 48 h. Afterward, LIVE/DEAD™ Viability/Cytotoxicity Kit (Thermo Fisher Scientific, MA, USA) was used to stain unviable cells. In the 2D cultures, the number of dead cells was quantified by counting red fluorescent objects, while in the 3D cultures, the mean intensity of red fluorescence was measured. Results were normalized to their respective untreated controls for both 2D and 3D cultures, allowing for the calculation of fold changes in cell death.

#### Statistical analysis

The normality of the samples was assessed with the Shapiro-Wilk test. Two-way ANOVA and Tukey’s comparison test were executed to compare the fraction of macrophages and genetic expression. Two-way ANOVA followed by Šídák’s multiple comparisons test was performed to compare cell death in 2D and 3D. Calculations were performed in GraphPad Prism 10.2.3 for Windows (GraphPad Software, Boston, Massachusetts, USA).

## Electronic supplementary material

Below is the link to the electronic supplementary material.

Supplementary Material 1

## Data Availability

The datasets analyzed during the study are available from the corresponding author upon request.
