# Supplementary material for: Development of tetraculture spheroids as a versatile 3D model for personalized breast cancer research
Source: Sci Rep. 2025 Jul 28;15:27449. doi: 10.1038/s41598-025-12556-9 (PMC12304226; doi:10.1038/s41598-025-12556-9)
Supplement: Supplementary file 1 — Supplementary Material 1 [file 41598_2025_12556_MOESM1_ESM.pdf]

Supplement Table 1. Sequences of utilized primers.

Gene	Forward	Reverse	Probe
B2M	ACCCTCACTATGTGGAGAACA	ATACCTGGGGCCATACACCT	SYBR
FN1	GACAGGAAAGAGATGCGCCA	CTCCCCTGTGCCATTCTCATA	SYBR
MMP2	ATGCCGCCTTTAACTGGAG	GGAAGCCAGGATCCATTTTC	No. 77
COL1A2	GGCAGTGATGGAAGTGTGG	CCAACAGCTCCAATTTCACC	No. 67

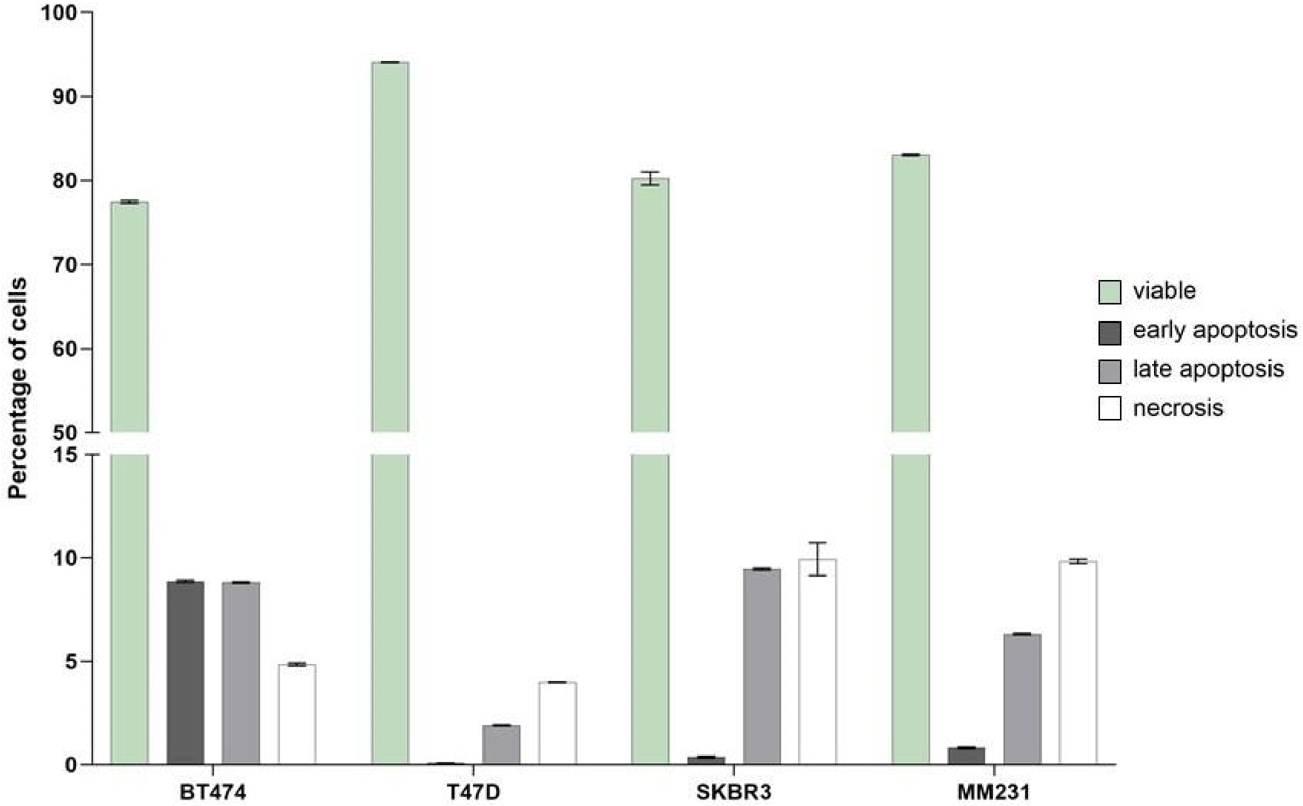

Supplement Figure 1. Percentage of viable, apoptotic and necrotic cells in 3D MCTSs. Spheroids were stained with Annexin V Apoptosis Detection Kits (Thermo Fisher Scientific, USA) and fractions of apoptotic, necrotic and viable cells were assessed by flow cytometry. Significant amount of cells within MCTSs were viable, with minor fraction of necrotic cells (up to 10% of all cells). T47D, SKBR3 and MDA-MB-231 MCTSs had very small fraction of

cell in the early apoptosis stage, whereas BT474 MCTSs had more significant amount of early apoptotic cells.

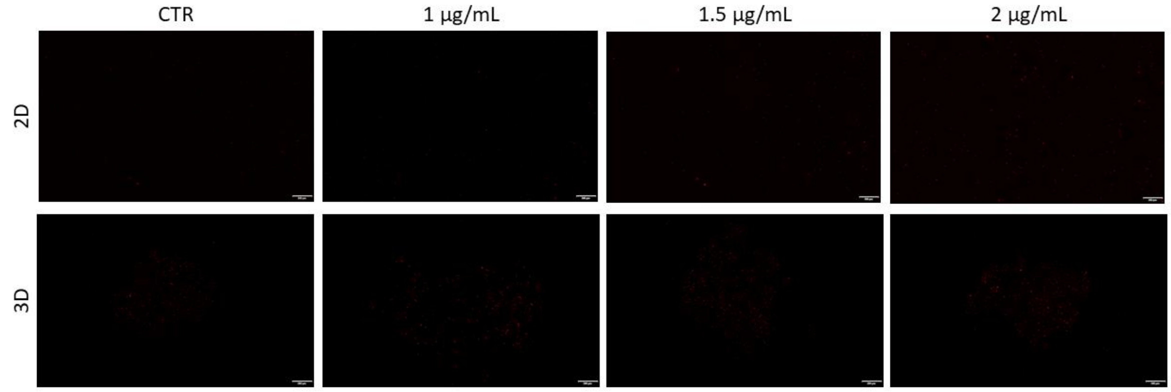

Supplementary Figure 2. Representative raw data images of 2D vs. 3D SKBR3 heterogenous cultures utilized for calculation of cell viability after cisplatin (1-2 μg/mL) treatment. LIVE/DEAD™ Viability/Cytotoxicity Kit (Thermo Fisher Scientific, MA, USA) was used to stain unviable cells. In the 2D cultures, the number of dead cells was quantified by counting red fluorescent objects, while in the 3D cultures, the mean intensity of red fluorescence was measured. Results were normalized to their respective untreated controls for both 2D and 3D cultures, allowing for the calculation of fold changes in cell death.

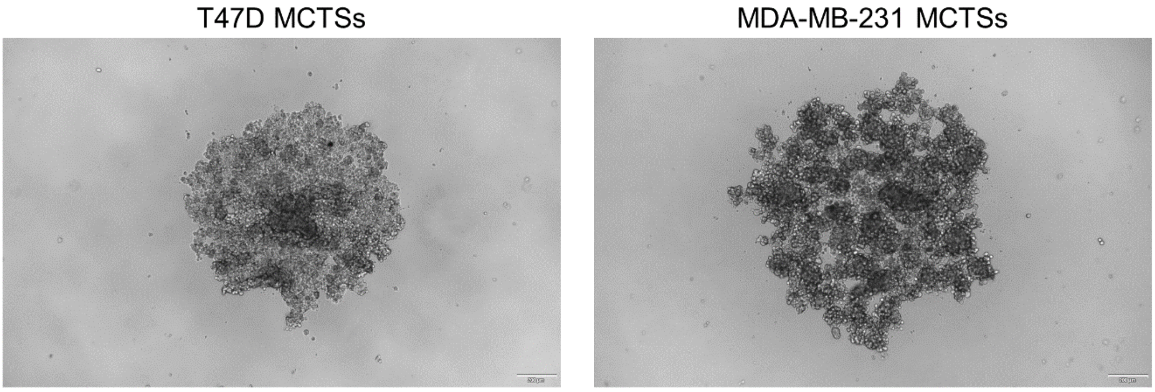

Supplement Figure 3. T47D and MDA-MB-231 MCTSs cultured in RPMI-1640 medium with supplements. Spheroids exhibit worse spheroid formation and form looser aggregates compared to when cultured in DMEM/F12.
